# Tamoxifen therapy benefit for patients with 70-gene signature high and low risk

**DOI:** 10.1007/s10549-017-4428-9

**Published:** 2017-08-04

**Authors:** Laura J. van ‘t Veer, Christina Yau, Nancy Y. Yu, Christopher C. Benz, Bo Nordenskjöld, Tommy Fornander, Olle Stål, Laura J. Esserman, Linda Sofie Lindström

**Affiliations:** 1 Department of Laboratory Medicine, University of California San Francisco, 2340 Sutter Street, San Francisco, CA 94115 USA; 2 Department of Surgery, University of California San Francisco, 1600 Divisadero Street, San Francisco, CA 94115 USA; 3 Buck Institute for Research on Aging, 8001 Redwood Boulevard, Novato, CA 94945 USA; 4 Department of Medicine, University of California San Francisco, 1600 Divisadero Street, San Francisco, CA 94115 USA; 5 Department of Biosciences and Nutrition, Karolinska Institutet, Hälsovägen 7, 141 83 Stockholm, Sweden; 6 Department of Clinical and Experimental Medicine and Department of Oncology, Linköping University, Sandbäcksgatan 7, 581 83 Linköping, Sweden; 7 Department of Oncology-Pathology, Karolinska Institutet, Z1:00, 171 76 Stockholm, Sweden

**Keywords:** 70-gene signature, Tamoxifen benefit, Endocrine therapy, Breast cancer, Long-term survival

## Abstract

**Background:**

Breast cancer molecular prognostic tools that predict recurrence risk have mainly been established on endocrine-treated patients and thus are not optimal for the evaluation of benefit from endocrine therapy. The Stockholm tamoxifen (STO-3) trial which randomized postmenopausal node-negative patients to 2-year tamoxifen (followed by an optional randomization for an additional 3-year tamoxifen vs nil), versus no adjuvant treatment, provides a unique opportunity to evaluate long-term 20-year benefit of endocrine therapy within prognostic risk classes of the 70-gene prognosis signature that was developed on adjuvantly untreated patients.

**Methods:**

We assessed by Kaplan–Meier analysis 20-year breast cancer-specific survival (BCSS) and 10-year distant metastasis-free survival (DMFS) for 538 estrogen receptor (ER)-positive, STO-3 trial patients with retrospectively ascertained 70-gene prognosis classification. Multivariable analysis of long-term (20 years) BCSS by STO-3 trial arm in the 70-gene high-risk and low-risk subgroups was performed using Cox proportional hazard modeling adjusting for classical patient and tumor characteristics.

**Results:**

Tamoxifen-treated, 70-gene low- and high-risk patients had 20-year BCSS of 90 and 83%, as compared to 80 and 65% for untreated patients, respectively (log-rank *p* < 0.0001). Notably, there is equivalent tamoxifen benefit in both high (HR 0.42 (0.21–0.86), *p* = 0.018) and low (HR 0.46 (0.25–0.85), *p* = 0.013) 70-gene risk categories even after adjusting for clinico-pathological factors for BCSS. Limited tamoxifen exposure as given in the STO-3 trial provides persistent benefit for 10–15 years after diagnosis in a time-varying analysis. 10-year DMFS was 93 and 85% for low- and high-risk tamoxifen-treated, versus 83 and 70% for low- and high-risk untreated patients, respectively (log-rank *p* < 0.0001).

**Conclusions:**

Patients with ER-positive breast cancer, regardless of high or low 70-gene risk classification, receive significant survival benefit lasting over 10 years from adjuvant tamoxifen therapy, even when given for a relatively short duration.

**Electronic supplementary material:**

The online version of this article (doi:10.1007/s10549-017-4428-9) contains supplementary material, which is available to authorized users.

## Introduction

Breast cancer is a diverse disease both in the sense of the metastatic potential of the primary tumor as well as time for metastasis to occur. The biological factors influencing the long-term risk of fatal breast cancer are unknown. It is, however, known that patients with newly diagnosed hormone receptor (HR)-positive breast cancer (ER− and/or progesterone (PR)-positive disease) have a continuous long-term risk for fatal breast cancer progression relative to stage-matched patients with newly diagnosed HR-negative cancer [1]. Endocrine therapy remains the cornerstone in management of HR-positive breast cancer since adjuvant use of either tamoxifen or aromatase inhibitors significantly improves the long-term survival of patients with either localized or regional HR-positive breast cancer [2, 3]. Survival benefit of adjuvant endocrine therapy has been shown to be independent of patient age, menopausal status, quantitative ER expression, nodal status, tumor size, grade, and proliferation rate [3, 4], and almost all newly diagnosed HR-positive breast cancers are nowadays treated with endocrine therapy. Nonetheless, this survival benefit varies markedly among similarly staged patients since up to half of all HR-positive patients receive little or no benefit from adjuvant endocrine therapy [3, 5], presumably due to breast cancer inter-tumor heterogeneity associated with endocrine resistance.

Gene expression signatures have helped us understand the inter-tumor heterogeneity between breast cancer tumors [6, 7], separating tumors into subgroups with different underlying biology, prognosis, and treatment benefit [8–10]. Unlike many other gene expression signatures, the 70-gene prognosis signature was developed on a systemically untreated patient cohort [11], which makes the signature suitable to evaluate treatment benefit.

Given the pressing need to understand more about long-term breast cancer survival and endocrine therapy benefit, we evaluated long-term endocrine therapy benefit in women with 70-gene low- and high-risk prognosis signatures using a large Swedish clinical trial (STO-3) having complete long-term (20 years) follow-up of patients randomized to receive adjuvant tamoxifen versus not.

## Methods

### The Stockholm tamoxifen (STO-3) trial

The Stockholm Tamoxifen (STO-3) trial enrolled postmenopausal patients with lymph node-negative breast cancers with tumors less than or equal to 30 mm in diameter, randomized to 2 years of adjuvant tamoxifen (40 mg daily) versus no adjuvant treatment. Patients in the tamoxifen arm, who were relapse free after 2 years of tamoxifen and who re-consented, were further randomized to 3 additional years of tamoxifen or none. From the original randomized trial cohort, 808 patients had formalin-fixed paraffin-embedded (FFPE) tissue blocks of primary breast cancer tumor available for molecular analyses, and of these, 81 patients were excluded because there was insufficient invasive tumor present for analysis. The remaining 727-patient subset with FFPE material available is well balanced to the original STO-3 cohort with regard to tumor characteristics, such as tumor size, ER status, and treatment arm assignment [12]. All patients included in the STO-3 randomized trial have detailed patient and clinical information. This study followed REMARK criteria [13].

In Sweden, all residents have a unique national registration number, which enables automatic linkage of various records of personal information from Regional and National registers of high validity and essentially complete coverage. Death due to breast cancer was assessed from the Swedish National Cause-of-Death Register with a reported accuracy of more than 96% from January 1, 1961 and onwards [14, 15]. The information on cause of death is from death certificates filled out by the treating physicians. Furthermore, information on contralateral breast cancer was assessed from the Swedish National Cancer Registry. Cancer registration has a legal basis in Sweden, and the Swedish Cancer Registry has a breast cancer coverage of more than 96% in validation studies [16]. Finally, information on distant metastasis was assessed from the Stockholm Breast Cancer Registry, a population-based registry held by the Regional Cancer Centre in Stockholm. The Breast Cancer Registry carries information on all breast cancer diagnoses in the Swedish counties of Stockholm and Gotland since 1976 as well as follow-up information including local relapse and distant metastasis.

The STO-3 trial was approved by the ethical committee at Karolinska Institutet, and participants provided oral consent.

#### 70-gene signature assignments

Gene expression data were independently generated using custom-designed arrays, Agilent Technologies (CA, USA), containing approximately 32.1K probes, representing approximately 21.5K unique genes from FFPE breast cancer tumor tissue. Approximately 90% (or 652 of 727 breast cancer tumors) passed the RNA quality check (according to the diagnostic quality model) and were used in the analysis. The 70-gene (MammaPrint) signature was performed according to standard protocols as previously described, including the use of 465 normalization genes and over 250 probes for hybridization and printing quality control. Patient tumor samples were classified into high or low risk by the 0.00 threshold in the MammaPrint index (high up to and low above 0.00 index, respectively). The subgroup of ultralow tumors is defined by MammaPrint index > +0.355 [17–19].

#### ER, PR, HER2, and Ki-67 immunohistochemistry

538 of the 652 tumors available for 70-gene evaluation were ER positive. Immunohistochemistry (IHC) was retrospectively done for ER, progesterone receptor [PR], human epidermal growth factor receptor 2 [HER2], and Ki-67 using DAKO Link48 Autostainer at the University of California Davis Medical Center (UCDMC). The antibodies used were ER (SP1; Spring Bioscience M301), PR (PgR 636; DAKO IR068), HER2 (HercepTest; DAKO SK001), and Ki67 (MIB-1; DAKO M7240), with EnVision + detection, following standard recommended procedures and with per-run positive controls assessed by quantitative image analysis to ensure consistent run-to-run staining intensity [19].

#### Tumor grade

Tumor grade according to the Nottingham system was retrospectively assessed by one pathologist [12].

### Statistical methods

#### Survival analyses

The outcome of interest was death due to breast cancer, and analyses of long-term breast cancer-specific survival (20 year) by the 70-gene risk classification (high and low risk) were performed in patients with ER-positive tumors. Patient follow-up started at the date of primary breast cancer diagnosis and ended at the date of death, contralateral breast cancer diagnosis, emigration from Sweden (only five women emigrated in total), or end of study follow-up (December 31, 2012).

For comparison with previous studies, we also performed 10-year analysis of distant metastasis-free survival. However, information on metastasis is less complete as compared to information on death. In our study, approximately 2%, i.e., 14 patients out of 727 patients, died from breast cancer but have missing information on metastasis. In patients with ER-positive disease and available gene expression information (538 patients) as included in this study, 11 patients who died from breast cancer had missing information on metastasis. For these 11 patients, date of death was used instead of the date of metastasis.

Kaplan–Meier analyses were performed by STO-3 trial arm and 70-gene risk classification. The significance was assessed using the log-rank test.

Multivariable analysis by the 70-gene risk classification was performed using Cox proportional hazard modeling adjusting for classical patient and tumor characteristics (age and calendar period of diagnosis, progesterone receptor status, HER2 status, Ki-67 status, tumor grade, and tumor size). Multivariable analysis for the ultralow 70-gene risk group by trial arm was not performed due to low sample size.

Flexible parametric survival models were used to estimate hazard ratios over time since diagnosis. Breast cancer-specific death rates were modeled through flexible parametric survival models using a restricted cubic spline function for the baseline mortality rate [20, 21]. Time-dependent multivariable analysis was performed for 1-, 5-, 10-, 15-, and 20-year follow-up time points, adjusting for the same patient and tumor characteristics as listed above. A spline with three degrees of freedom was used to estimate the hazard ratios. For the time-dependent covariate (tamoxifen trial arm), we used a second spline function with one degree of freedom to model the interactions between the covariate and time. The stpm2 function in Stata version 14.2 was used for the modeling and the analyses [20].

The proportional hazard assumption for the main exposure variable (70-gene risk classification) was assessed by including a time-dependent covariate in the model. No significant deviation was noted. Data preparation and analysis were done using SAS version 9.4, Stata version 14.2, and R version 3.4.0.

## Results

Patients in the STO trial with ER-positive breast cancer disease and available 70-gene expression signature data, 538 patients in total, were included in our analysis. In Table 1, patient and tumor characteristics by the 70-gene risk classification (high versus low risk) are presented. Of 167 patients with tumors classified as being high risk (167/538, 31%), 54% of the tumors were PR positive, 14% were HER2 positive, 41% had Ki-67 greater than 15, and 5.5% of tumors were grade 1. Of the 371 patients with tumors classified as being low risk (371/538, 69%), 76% of the tumors were PR positive, no tumors were HER2 positive, 15% had Ki-67 greater than 15, and 29% of tumors were grade 1.

**Table 1:** Patient and tumor characteristics by 70-gene risk classification

	STO-3 trial				
	70-Gene high risk		70-Gene low risk		Total number of patients
	Number	Percent	Number	Percent	
STO-3 trial arm					
Tamoxifen-treated arm	82	49.1	199	53.6	281
Untreated arm	85	50.9	172	46.4	257
Patient characteristics					
Calendar period of primary diagnosis					
1976–1984	92	55.1	182	49.1	274
1985–1990	75	44.9	189	50.9	264
Age at primary diagnosis (years)					
45–54	22	13.2	30	8.1	52
55–64	81	48.5	184	49.6	265
65–74	64	38.3	157	42.3	221
Primary tumor characteristics					
Type of surgery					
Breast-conserving surgery and RT	28	16.8	93	25.1	121
Mastectomy	139	83.2	278	74.9	417
Progesterone receptor status					
Positive	89	53.9	278	76.4	367
Negative	76	46.1	86	23.6	162
Unknown	2	–	7	–	9
HER2 status^a^					
Positive	24	14.4	0	0	24
Negative	143	85.6	370	100	513
Unknown	0	–	1	–	1
Ki-67 status^b^					
Positive	66	41.3	51	14.5	117
Negative	94	58.7	301	85.5	395
Unknown	7	–	19	–	26
Tumor grade					
1	9	5.5	107	29.2	116
2	91	55.5	247	67.5	338
3	64	39.0	12	3.3	76
Unknown	3	–	5	–	8
Tumor size					
pT < 20 mm	122	74.4	315	85.6	437
pT ≥ 20 mm	42	25.6	53	14.4	95
Unknown	3	–	3	–	6

^a^HER2 positive defined as 3+ by immunohistochemistry

^b^Ki-67 cut-off for positivity at 15%

### Survival analysis

#### Univariate survival analysis

**Fig. 1:**
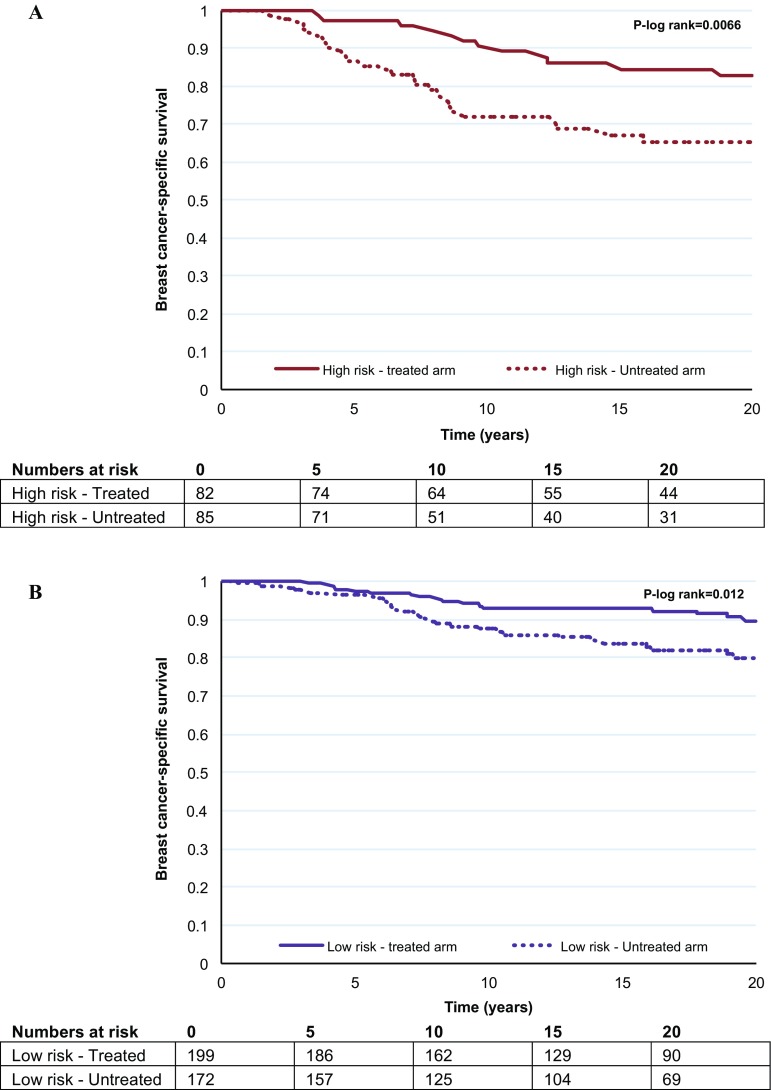
Kaplan–Meier analysis of breast cancer-specific survival by 70-gene risk classification and trial arm (tamoxifen treated versus untreated). The *p* value is based on the log-rank test, and numbers at risk are shown underneath the graph. **a** 70-gene high risk by trial arm (with and without tamoxifen). **b** 70-gene low risk by trial arm (with and without tamoxifen)

Kaplan–Meier survival graphs for patients with and without tamoxifen are shown per trial arm and by the standard 70-gene high- and low-risk groups in Fig. 1S. A statistically significant difference in long-term (20 year) breast cancer-specific survival by STO-3 trial arm and 70-gene classification was observed (log-rank *p* < 0.0001). For the 70-gene low-risk group, the 20-year breast cancer-specific survival with and without tamoxifen treatment was 90% (95% CI 84–94%) and 80.0% (95% CI 72–86%), respectively. For the 70-gene high-risk group, the 20-year breast cancer-specific survival with and without tamoxifen treatment was 83% (95% CI 72–90%) and 65% (95% CI 53–75%), respectively.

**Fig. 2:**
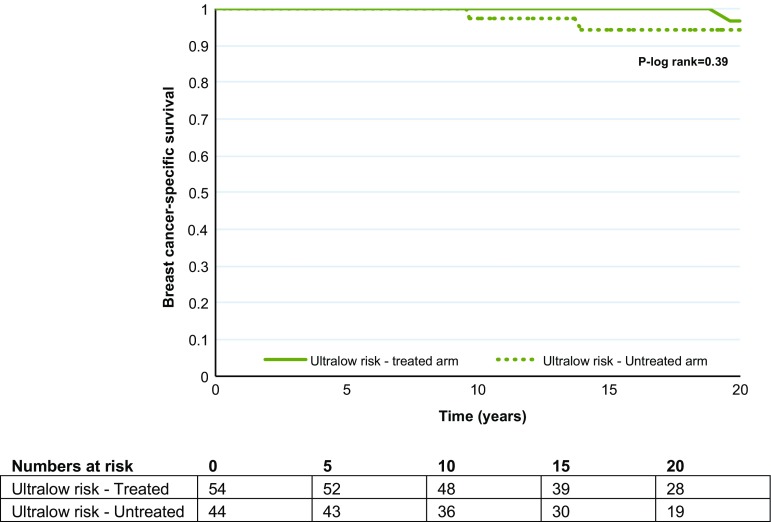
Kaplan–Meier analysis of breast cancer-specific survival by 70-gene ultralow risk and trial arm. The *p* value is based on the log-rank test, and numbers at risk are shown underneath the graph

The benefit of tamoxifen within each 70-gene risk group is shown in Fig. 1a and b, respectively. A statistically significant difference in long-term (20 year) breast cancer-specific survival by STO-3 trial arm (tamoxifen-treated versus untreated) was seen within the high- as well as the low-risk group (70-gene high risk, tamoxifen yes/no: log-rank *p* = 0.0066, 70-gene low risk, tamoxifen yes/no: log-rank *p* = 0.012). The benefit of tamoxifen was further investigated in the earlier defined ultralow-risk group of breast cancer patients with indolent disease and extremely good outcome [19]. No statistically significant difference in long-term survival was seen (Fig. 2, log-rank *p* = 0.39) in the ultralow-risk group for patients receiving tamoxifen therapy or not.

In order to compare results to previous studies that evaluated endocrine treatment benefit in prognostic high- and low-risk subgroups up to 10 year after diagnosis, we also performed a 10-year analysis of distant metastasis-free survival by the 70-gene risk classification using Kaplan–Meier analysis (four groups: low risk/treated arm, low risk/untreated arm, high risk/treated arm, and high risk/untreated arm). A statistically significant difference in 10-year distant metastasis-free survival was seen (Fig. 2S log-rank, *p* < 0.0001). For the 70-gene low-risk group, the 10-year distant metastasis-free survival with and without tamoxifen treatment was 93% (95% CI 88–96%) and 83% (95% CI 76–88%), respectively. For the 70-gene high-risk group, the 10-year distant metastasis-free survival with and without tamoxifen treatment was 85% (95% CI 75–91%) and 70% (95% CI 58–79%), respectively.

#### Multivariable survival analysis

Multivariable analysis of long-term (20 years) breast cancer-specific survival by STO-3 trial arm in the 70-gene high-risk and the low-risk subgroups was performed using Cox proportional hazard modeling adjusting for classical patient and tumor characteristics (age and calendar period of diagnosis, progesterone receptor status, HER2 status, Ki-67 status, tumor grade, and tumor size). Interestingly, both patients classified as 70-gene high, as well as low risk notably benefited from tamoxifen treatment (Table 2) after adjusting for clinico-pathological factors. Patients with high-risk tumors that were in the tamoxifen trial arm had a significantly reduced risk of dying from breast cancer, relative risk reduction of 58%, as compared to patients in the untreated arm [Hazard ratio (HR), 0.42; 95% CI 0.21–0.86]. Low-risk patients that were treated with tamoxifen had a significantly reduced risk (relative risk reduction of 54%) of dying from breast cancer [Hazard ratio (HR), 0.46; 95% CI 0.25–0.85].

**Table 2:** Risk of long-term breast cancer-specific death (20 year) by 70-gene classification and trial arm in ER-positive breast cancer

STO-3 trial	STO-3 trial arm		Breast cancer-specific deaths^a^	Breast cancer-specific survival^a^	
Patients included	Trial arm	Number		HR (95% CI)	*p* value (*χ* ^2^)
70-gene High risk					
Adjusting for classical patient and tumor characteristics^£^	Treated arm^b^	82	12	**0.42 (0.21–0.86)**	**0.018 (5.62)**
	Untreated arm	85	26	1.0 ref.	
70-gene Low risk					
Adjusting for classical patient and tumor characteristics^£^	Treated arm^b^	199	17	**0.46 (0.25–0.85)**	**0.013 (6.15)**
	Untreated arm	172	29	1.0 ref.	

Hazard rates in bold indicate statistically significant values

^a^20-year breast cancer-specific survival

^b^Modeled by multivariable proportional hazard (Cox) analyses adjusting for age and calendar period of diagnosis, progesterone receptor status, HER2 status, Ki-67 status, tumor grade, and tumor size

Finally, time-dependent multivariable analyses were also performed 1, 5, 10, 15, and 20 years after breast cancer diagnosis using flexible parametric survival models to estimate hazard ratios. For both low- and high-risk tumors, patients had a significant benefit of tamoxifen treatment up to 10 years after diagnosis but less benefit after (Table 3).

**Table 3:** Time-varying analysis of the long-term risk for breast cancer-specific death (20 years) by 70-gene classification and trial arm in ER-positive breast cancer

STO-3 trial	STO-3 trial arm		Breast cancer-specific deaths^a^	Years since diagnosis	HR (95% CI)
Patients included	Trial arm	Number			
70-gene High risk^b^	Treated arm	82	12	**1**	**0.20 (0.05–0.74)**
				**5**	**0.37 (0.21–0.67)**
				**10**	**0.55 (0.33–0.90)**
				15	0.81 (0.33**–**1.99)
				20	1.00 (0.31**–**3.28)
	Untreated arm	85	26	1.0 ref.	
70-gene Low risk^b^	Treated arm	199	17	**1**	**0.20 (0.05–0.75)**
				**5**	**0.37 (0.20–0.67)**
				**10**	**0.53 (0.3–0.87)**
				15	0.75 (0.35**–**1.62)
				20	0.90 (0.33**–**2.84)
	Untreated arm	172	29	1.0 ref.	

Hazard rates in bold indicate statistically significant values

^a^20-year breast cancer-specific survival

^b^Modeled by flexible parametric survival analysis adjusting for age and calendar period of diagnosis, progesterone receptor status, HER2 status, Ki-67 status, tumor grade, and tumor size

## Discussion

In this study, we had the rare opportunity to observe the long-term (20-year) impact of tamoxifen therapy versus not as the sole adjuvant therapy in women whose tumors were retroactively molecularly classified as either 70-gene high or low risk. The results demonstrate that there is a significant and comparable risk reduction benefit from tamoxifen in both groups, and that the survival benefit after 2 years of tamoxifen use (for one-third of patients in the tamoxifen arm after 5 years) continues for well over 10 years. These findings also confirm what has previously been shown, that the 70-gene classification is prognostic, and that women with low risk versus high risk have higher survival independent of treatment. In this study, adjuvant tamoxifen appeared to reduce risk of death by 50%, regardless of 70-gene high- or low-risk biology.

It has been shown that breast cancer stage and grade do not appreciably affect the proportional risk reduction benefit from 5 years of adjuvant tamoxifen therapy [3]; this study, however, represents the first comparative analysis of adjuvant tamoxifen’s risk reduction benefit based on a priori molecularly defined risk categorization, specifically the 70-gene prognosis signature low- and high-risk subgroups. These STO-3 outcome data also serve to remind us about the natural history of HR-positive breast cancer and illustrate the very long tail of death from breast cancer. Even, those women with low-risk tumor biology continue to have risk of death, and in fact their risk of death is higher after 5 years and this continues for at least 20 years. For those women with molecularly high risk (by the 70-gene classification), their recurrence risk also persists up to 20 years, but the bulk of the risk is experienced in the first 5 or (to lesser extent) 10 years. Interestingly, the benefit of adjuvant tamoxifen treatment shows the same proportional reduction of risk of death over time for both low- and high-risk groups. In contrast to this observation, for those STO-3 patients for whom we recently defined having a 70-gene indolent/ultralow-risk disease with an extremely low 20-year risk of death [19], we show that BCSS benefit from adjuvant tamoxifen does not achieve clinical significance over a period of 20 years relative to untreated patients with ultralow-risk ER-positive breast cancer.

Most prognostic tools have been shown to determine risk of recurrence only out to 10 years in a uniformly endocrine-treated population, making these tools less suitable to evaluate their ability to identify which patients might and to what extent benefit from endocrine therapy [3]. This is the first time a prognostic tool shows a benefit of tamoxifen as the sole adjuvant therapy for molecularly identified high-risk patients, alongside the benefit in low-risk patients. The reason that the 70-gene signature is able to identify this benefit across all risk groups is based on the fact that the signature was developed in an adjuvant treatment-naïve population, whereas other prognosis signatures like Oncotype and Endopredict have been developed on tamoxifen-treated population [9, 22]. The benefit of tamoxifen is therefore difficult to discern [9]. Our analysis of distant metastasis-free survival at 10 years also allows direct comparison with previously published papers for patients having received endocrine treatment which results in comparable 10-year DMFS rates [9, 22, 23].

Interestingly, this paper also likely confirms the additive benefit of chemotherapy, as risk for recurrence is more substantial in the first 5 years in the high-risk group as compared to the low-risk group. Even if the relative benefit of tamoxifen risk reduction is the same, the absolute risk of recurrence is higher in the high-risk group, where chemotherapy is known to exert its optimal effect [24]. In the low-risk and particularly the ultralow-risk group, the absolute risk of recurrence is extremely low in the first 5 years, therefore making it unlikely that there would be benefit from chemotherapy, an observation recently made in the large prospective randomized MINDACT trial for breast cancers with 70-gene low-risk signature even within the clinical high-risk setting [10, 25].

In order to advance the science of personalized medicine, diagnostics need to help us determine who will benefit and when. Women with ultralow-risk profiles have almost no risk and therefore benefit little if at all even from endocrine treatment. Women with biologically high-risk disease benefit from tamoxifen, but have high early residual risk supporting a decision to intercede with chemotherapy. Women with low- but not ultralow-risk disease, as well as those with high-risk disease, would be very well served if we had a robust marker of sensitivity to endocrine therapy and chemotherapy, to understand their long-term risk to die from breast cancer. That way, we could find ways to intercede for this group of women, and, as well, to determine for whom endocrine therapy is not sufficient. In order to more rapidly advance the field, we have to be able to identify those women at diagnosis and specifically focus more targeted interventions. Establishing the long-term benefit of endocrine therapy in prognostic subclasses as described here contributes to our ability to guide the use of adjuvant therapy in breast cancer.

## Electronic supplementary material

Below is the link to the electronic supplementary material.

Supplementary material 1 (DOCX 108 kb)
