# Supplementary material for: Tamoxifen therapy benefit for patients with 70-gene signature high and low risk
Source: Breast Cancer Res Treat. 2017 Aug 4;166(2):593–601. doi: 10.1007/s10549-017-4428-9 (PMC5668340; doi:10.1007/s10549-017-4428-9)
Supplement: Supplementary file 1 — Supplementary material 1 (DOCX 108 kb) [file 10549_2017_4428_MOESM1_ESM.docx]

**Figures**

**Tamoxifen therapy benefit for patients with 70-gene signature high and low risk**

Laura J. van 't Veer,^1,*^ Christina Yau,^2,3^ Nancy Y. Yu,^5^ Christopher C. Benz, ^3,4^ Bo Nordenskjöld,^6^ Tommy Fornander,^7^ Olle Stål,^6^ Laura J. Esserman,^2^ and Linda S. Lindström^5,*^

**Figure 1S** Kaplan Meier analyses of breast cancer-specific survival (20 year) by 70-gene risk classification (high and low risk) and trial arm (four groups: high risk/treated arm, high risk/untreated arm, low risk/treated arm, low risk/untreated arm). The P-value is based on the Log-rank test, numbers at risk are shown underneath the graph.

| **Numbers at risk** | **0** | **5** | **10** | **15** | **20** |
| --- | --- | --- | --- | --- | --- |
| Low risk - Treated | 199 | 186 | 162 | 129 | 90 |
| Low risk - Untreated | 172 | 157 | 125 | 104 | 69 |
| High risk - Treated | 82 | 74 | 64 | 55 | 44 |
| High risk - Untreated | 85 | 71 | 51 | 40 | 31 |

**Figure 2S** Kaplan Meier analyses of distant metastasis-free survival (10 year) by the 70-gene risk classification (high and low risk) and trial arm (four groups: high risk/treated arm, high risk/untreated arm, low risk/treated arm, low risk/untreated arm). The P-value is based on the Log-rank test, numbers at risk are shown underneath the graph.

| **Numbers at risk** | **0** | **5** | **10** | KM estimate  (95% CI) |
| --- | --- | --- | --- | --- |
| Low risk - Treated | 199 | 180 | 159 | 0.93 (0.88-0.96) |
| Low risk - Untreated | 172 | 146 | 116 | 0.83 (0.76-0.88) |
| High risk - Treated | 82 | 71 | 57 | 0.85 (0.75-0.91) |
| High risk - Untreated | 85 | 63 | 48 | 0.70 (0.58-0.79) |
